# Improving the Social Relevance of Experimental Stroke Models: Social Isolation, Social Defeat Stress and Stroke Outcome in Animals and Humans

**DOI:** 10.3389/fneur.2020.00427

**Published:** 2020-05-14

**Authors:** Chloe A. Lowry, Albert Y. Jin

**Affiliations:** ^1^Centre for Neuroscience Studies, Queen's University, Kingston, ON, Canada; ^2^Department of Medicine, Queen's University, Kingston, ON, Canada; ^3^Department of Biomedical and Molecular Sciences, Queen's University, Kingston, ON, Canada

**Keywords:** stroke, social determinants of health, social isolation, social defeat stress, inflammation

## Abstract

The outcome of ischemic stroke varies across socioeconomic strata, even among countries with universal health care. Emerging evidence suggests that psychosocial aspects of low socioeconomic status such as social isolation and social defeat stress interact with, and contribute to, stroke pathophysiology. However, experimental investigations of stroke rarely account for such socioeconomic influences. Social isolation in stroke survivors is associated with increased infarction volume, increased risk of post-stroke depression, and worse long-term functional outcome. Social defeat is thought to contribute significantly to chronic stress in low socioeconomic status groups and is associated with poor health outcomes. Chronic stress is also associated with worse post-stroke functional outcome and greater disability even after accounting for stroke severity, vascular risk factors, and access to acute stroke care. Experimental stroke studies which incorporate social isolation or social defeat stress have shown that both tissue and functional stroke outcome is affected by the increased expression of TNF-α and IL-6, increased glucocorticoid production, and suppression of the protooncogene *bcl-2*. This review explores the consequences of social isolation and social defeat stress on stroke, preclinical stroke models that have been used to investigate these factors, and possible molecular mechanisms underlying the influence of socioeconomic disparities on stroke outcome.

## Introduction

Stroke remains a leading cause of death and adult disability worldwide, presenting an enormous societal burden (1) for which there are limited treatments. Indeed, over one thousand potential neuroprotectant compounds have shown efficacy in animals but have failed in clinical trials (2). While the reason for this translational failure is multi-faceted, a common criticism of stroke research is that preclinical animal models of stroke do not adequately capture the characteristics of the clinically targeted population (3). While clinical stroke outcome is influenced by a myriad of factors including initial stroke severity, stroke subtype, age, sex, co-morbidities, polypharmacy, and access to rehabilitation (4), a growing body of literature suggests that factors associated with low socioeconomic status (SES) contribute to stroke pathophysiology (5). This review will examine the influence of social isolation and stress from social defeat (i.e., losing or becoming subordinate as the outcome in social conflict or competition) on stroke pathophysiology and outcome, and how preclinical stroke models might incorporate these important factors in the development of new stroke therapeutics.

## Socioeconomic Status, Social Isolation and Social Defeat Stress: Consequences for Stroke

Low SES negatively influences nearly all social determinants of health, and is inextricably linked to disease outcomes and a variety of health conditions (6–8). Low SES is not simply a lack of money, but also includes insufficient access to various necessities of life, including housing, health care and other resources (9). Low SES has been shown to influence morbidity and mortality of stroke worldwide (5), increase the incidence of stroke, and affect accessibility of evidence-based stroke care in both the acute and chronic stages of injury—even among countries with universal health care (5, 10, 11). Importantly, acute and long-term functional outcome following stroke appears to be linked to SES independent of stroke severity, risk factors, and access to acute stroke care (12–14). The specific aspects of low SES which affect stroke outcome remain to be clarified, but social isolation and social defeat stress may have an important influence on both stroke injury and stroke recovery.

Social isolation (SI) is the condition in which people have few personal contacts (typically three or less) amongst their friends, families and neighbors (15). SI predicts morbidity and mortality from a variety of diseases and conditions (16, 17), is a known risk factor for Alzheimer's disease (18), and can have detrimental effects on the outcome of other neurological disorders, including Parkinson's disease (19) and multiple sclerosis (20). SI is a particularly potent risk factor for poor stroke outcome (21–23), compounded by life-altering sequelae such as loss of mobility, dysphagia, cognitive impairment, aphasia, and visual disabilities (24). As a consequence many report feelings of isolation or fear, becoming socially withdrawn, increasingly housebound and disengaged from the recovery process (25). Additionally, stroke incidence and SI increase with age, resulting in a high prevalence of post-stroke depression (26), further contributing to social withdrawal and poor cognitive and functional outcomes (26, 27). Fortunately, social support systems and engagement have been shown to be protective for stroke survivors (21, 25). Social support was linked to a faster recovery rate, improved outcome (21) and, when facilitated by stroke survivor groups, decreased loneliness, increased feelings of empowerment and acceptance, and improved social competence (25).

Despite the well-recognized negative effects of low SES on disease incidence and outcome (6–8), preclinical modeling of low SES remains a challenge. While material deprivation can be replicated in animals through food or bedding restriction, other socioeconomic factors associated with poverty such as standard of living, neighborhood safety, and self-perception of social rank are more uniquely human. Chronic stress appears to be one mechanism through which low SES can influence health and disease (6, 28, 29). Individuals and families of lower SES experience increased stress (8, 30, 31) and low social rank is a profound stressor across many species, including humans (29). Chronic stress is associated with poor outcomes in a variety of neurological diseases, including Parkinson's disease, multiple sclerosis, dementia, and stroke (32, 33). Low SES is also associated with mental and physical stress as well as increased odds of intracranial atherosclerotic disease, a well-known stroke etiology (34). Kondo et al. demonstrated that poor health outcomes are more associated with people's perceptions of social deprivation and unfavorable social comparisons (for example, to co-workers, parents at a similar stage in life, or neighbors) rather than absolute income (35). Wood et al. found that objectively measured low social rank and the associated cognitions (defeat, entrapment) also seem to be a cause for mental distress rather than absolute income (36). This is possibly mediated through an “involuntary defeat syndrome” (37), analogous to the coping strategies used by animals faced with social defeat. Social status, where social position was assessed by the respondent's rating of where they stood within the social hierarchy, has also been linked to worse health outcomes (38). Therefore, the role of mental stress, and in particular social defeat stress as part of the spectrum of challenges faced by those with low SES, seems to be significant in health outcome, vascular risk factors, and increased vulnerability to stroke (23).

## Social Isolation and Social Defeat Stress in Animal Models of Stroke

### Social Isolation in Preclinical Models of Stroke

SI is a known stressor across many species (39), and can be defined as a near-complete or total lack of contact between members of a social species (40). In adult rodents, SI has been shown to elicit anxiety and depressive-like behaviors (41, 42) and the effects of actual or perceived isolation in humans can be modeled in rodents by utilizing a SI protocol (43). These paradigms vary in length, and may involve isolation rearing (i.e., isolation of pups at the time of weaning) or separation of adult mice from a group-housed environment later in the lifespan (41, 44).

Several studies have investigated the impact of SI on stroke outcome in rodents (17, 39, 43, 45, 46). In all cases, SI was shown to have detrimental effects on the extent of ischemia-mediated damage, functional outcome, and/or survival (17, 39, 43, 45, 46). While the precise mechanisms underlying this phenomenon have yet to be fully elucidated, it is evident that socially-deprived animals mount a distinct pathological response to ischemic injury compared to their socially-housed counterparts (17, 39, 45). Craft et al. showed that male and female mice housed singly both before and after middle cerebral artery occlusion (MCAO) had larger infarcts and elevated intra-ischemic serum concentration of C-reactive protein (CRP, a marker of systemic inflammation) compared to animals that were pair-housed (17). Additionally, isolated mice had worse functional outcomes than pair-housed mice, as evidenced by a significant decrease in contralateral paw use. Weil and colleagues demonstrated that SI of mice before and after global ischemia exacerbated ischemia-induced neuronal damage (45). Socially isolated mice showed a heightened inflammatory response in the hippocampus, characterized by increased microglial activation, and increased gene expression of the proinflammatory cytokine tumor necrosis factor-α (TNF-α). TNF-α is a primary mediator of the early immune response in both the brain and the periphery (47), and can activate a multitude of signaling cascades that ultimately lead to apoptosis or necrosis (48). By contrast, group-housed mice showed a near complete attenuation of the post-ischemic inflammatory reaction, possibly explaining the reduced neuronal damage observed in these animals. The effect of housing conditions on stroke outcome and survival was further demonstrated by Karelina et al. (39). Mice that were individually housed prior to and following MCAO had higher post-stroke serum concentration of interleukin-6 (IL-6, a pro-inflammatory cytokine), greater mortality rate at 7 days post-reperfusion (60%, compared to 0% for socially housed animals), and significantly increased infarct volume and cerebral edema. Administration of an IL-6 neutralizing antibody to both singly- and pair-housed mice prior to ischemia onset resulted in infarct sizes that were comparable between groups, thereby mitigating the influence of housing conditions on stroke outcome. Interestingly, housing conditions did not appear to influence functional outcomes (e.g., total locomotor activity, exploratory behavior, contralateral paw use), although behavioral metrics were conducted at earlier timepoints than in Craft et al. (72 h vs. 7 days). Consistent with these studies, Venna et al. showed that mice housed with either a healthy or stroked partner following MCAO had significantly smaller infarcts at 72 h post-stroke compared to isolated mice (46). Isolated mice also had significantly elevated serum IL-6 concentrations relative to socially housed animals. Even when pair housing was initiated 72 h after stroke, socially housed mice showed improved functional recovery (e.g., increased contralateral paw use and mobility on the tail suspension test) and decreased mortality compared to isolated animals, despite similar histological damage in all groups (46). The authors postulate that the improved functional recovery of pair-housed mice may be due to increased neurogenesis and BDNF levels seen in these animals compared to their isolated counterparts.

These findings in experimental stroke models may shed light on the effect of SI on stroke outcome in people. In humans, CRP is associated with the degree of social integration, with increased CRP in older men (>60 years) who have fewer social ties (49). A heightened peripheral inflammatory response—specifically, peak plasma IL-6, and CRP concentrations—is correlated with increased infarct volume at 7 days post-stroke, as well as worse functional outcome at 3 months (50). Future research should continue to characterize these mechanisms, as it may yield important insight into whether interventions aimed at reducing SI in the acute care setting and beyond can improve functional outcome for patients identified as having low levels of social support.

### Social Defeat Stress in Preclinical Models of Stroke

Chronic stresses associated with social position can adversely affect the hypothalamic-pituitary-adrenocortical (HPA) axis, the body's primary neuroendocrine mechanism for mounting a stress response, with prolonged activation associated with increased cardiovascular risk, increased susceptibility to infection, and immune suppression (28). Since stroke recovery also appears to be linked to the stress associated with SES (12, 14), utilizing a model of chronic social defeat stress in rodents may be one way to approximate the complex effects of social defeat stress on stroke outcome in humans.

Chronic social defeat stress is a common model for social dominance and subordination in rodents (29). This paradigm usually involves placing an “intruder” mouse of one strain into the cage of an aggressive “resident” mouse of a different strain (e.g., C57BL/6 “intruder” vs. CD-1 “resident”) (51). The resident mouse typically attacks the intruder one or more times, after which the two mice are separated by a transparent screen such that visual and auditory threats can continue until the intruder is returned to its home cage. The social defeat paradigm is repeated for a week or more, at which point the subordinated mouse shows depressive-like features and other signs of stress (e.g., social avoidance, decreased grooming) (52). Chronic social defeat stress repeatedly activates the HPA axis with each instance of social conflict (53). Thus, animal models of social defeat stress may be more relevant to the psychological stressors associated with low SES such as feelings of powerlessness.

The harmful impact of social defeat stress on experimental stroke injury has been previously investigated (32, 33). Sugo and colleagues exposed mice to social intimidation stress for 1 week prior to MCAO (33). Mice who were socially stressed or administered exogenous corticosterone prior to stroke had infarct volumes twice as large as those seen in unstressed and mifepristone-injected (a glucocorticoid receptor antagonist) groups. Additionally, both stressed and corticosterone-injected mice showed increased cognitive impairment post-stroke compared to unstressed and mifepristone-injected mice, as indicated by a decreased latency to cross in a passive avoidance task. The authors concluded that histological and functional outcome following stroke was compromised by chronic social stress, and was likely mediated through the action of corticosterone. Indeed, glucocorticoids are thought to potentiate post-ischemic neuronal morphological damage in the hippocampus and neocortex of rodents (47, 54), and high serum levels of cortisol are predictive of poor functional outcome, cognitive dysfunction, and mortality in stroke patients (55–57).

Another possible mechanism underlying the deleterious effects of stress on stroke outcome is through the protooncogene *bcl-2*. Elevated *bcl-2* expression in various neurodegenerative disorders is known to be protective against apoptosis and necrosis, and up-regulation of *bcl-2* in the ischemic penumbra occurs during stroke (32). In a study by DeVries et al. mice were exposed to chronic social defeat stress using a resident-intruder paradigm for 3 days prior to MCAO (32). Interestingly, *bcl-2* mRNA expression in the ischemic hemisphere of mice previously exposed to social stress was 70% lower compared to unstressed mice (32). Furthermore, stressed mice had infarct volumes four times as large as unstressed mice, and infarct size was significantly correlated with post-ischemic serum corticosterone concentration. The harmful effects of social intimidation stress on ischemic injury were abolished in transgenic mice that constitutively express higher levels of neuronal *bcl-2*, suggesting that *bcl-2* is an endogenous neuroprotective mechanism that is sensitive to the effects of psychogenic stress.

While the complex spectrum of challenges associated with low SES is difficult to model in rodents, utilizing social defeat stress combined with SI protocols may provide a reasonable proxy for understanding why individuals of lower SES are at risk for greater injury and poor functional outcome following stroke. This requires a more comprehensive experimental approach to account for psychosocial influences on stroke outcome. As it remains unclear if SI and social defeat stress are independent of one another, experimental design of preclinical studies should allow for assessment of these factors separately as well as in combination, in both the pre- and post-stroke environments. This will invariably require larger scale studies with multiple experimental cohorts. For example, animal groups could include SI pre- and post-stroke, SI in the pre- but not post-stroke environment, SI in the post- but not pre- stroke environment, SI + social defeat stress in the pre- and post- stroke environment, etc. This may help to elucidate the molecular bases of each stressor on stroke outcome, and assess any interaction between these two important aspects of low SES. In terms of quantifying psychosocial factors, typical measures of SI in people using subjective rating scales are not possible in animals. However, downstream effects of SI such as anxiety and depression (41, 42) can be quantified using the sucrose intake test, tail suspension test, forced swim test, and light-dark box (51, 52, 58). Social defeat and hierarchies in rodents that would be akin to those in humans can be measured using the tube dominance test (59) or social interaction test (51). As SI and social defeat stress often co-occur in people of low SES, accounting for these factors may improve the translational relevance of preclinical stroke models.

## Conclusion

SI and social defeat stress are two factors which are known to have deleterious effects on stroke outcome and can be modeled experimentally using SI and social defeat stress protocols. The molecular bases of the effects of SI and social defeat stress on short- and long-term stroke outcomes are still incompletely understood, although likely multifactorial. Increased inflammation and HPA axis activation, as well as suppression of *bcl-2* have emerged as possible mechanisms by which these social conditions mediate ischemic damage. Future investigations should continue to delineate these effects in order to identify novel therapeutic strategies aimed at mitigating stroke damage in both the acute and chronic stages of injury.

Utilizing preclinical models of stroke that more closely resemble the social complexity of the clinical population may improve the translational success of neuroprotectant therapies. Importantly, an enhanced understanding of how various social determinants of health interact with and contribute to stroke pathophysiology on a biochemical level may allow for the discovery of biomarkers which could be used to identify at-risk patients upon hospital admission. Such patients could then be proactively targeted with personalized pharmacological, social or community-based interventions to improve stroke outcomes not only in the hospital emergency room and stroke ward, but also during rehabilitation and the patient's return to their community.

## Author Contributions

CL was the primary researcher and author of the manuscript. AJ assisted with writing and editing the manuscript.

## Conflict of Interest

The authors declare that the research was conducted in the absence of any commercial or financial relationships that could be construed as a potential conflict of interest.
